# Urethrovaginal fistula 11 years after a bone anchor sling using woven polyester and treatment with a Martius flap

**DOI:** 10.1002/iju5.12374

**Published:** 2021-09-23

**Authors:** Kumiko Kato, Akitaka Suzuki, Yuji Hayashi, Aika Matsuyama, Hiroki Sai, Akinobu Ishiyama, Takashi Kato, Satoshi Inoue, Hiroki Hirabayashi, Shoji Suzuki

**Affiliations:** ^1^ Departments of Female Urology Japanese Red Cross Aichi Medical Center Nagoya Daiichi Hospital Nagoya Japan; ^2^ Department of Urology Japanese Red Cross Aichi Medical Center Nagoya Daiichi Hospital Nagoya Japan; ^3^ Department of Urology National Hospital Organization Nagoya Medical Center Nagoya Japan; ^4^ Department of Plastic Surgery Japanese Red Cross Aichi Medical Center Nagoya Daiichi Hospital Nagoya Japan

**Keywords:** bone anchor sling, Martius flap, polyester, stress urinary incontinence, urethrovaginal fistula

## Abstract

**Introduction:**

We encountered a urethrovaginal fistula diagnosed 11 years after a bone anchor sling.

**Case presentation:**

A 58‐year‐old woman underwent a bone anchor sling to treat stress urinary incontinence. At age 69, mid‐urethral sling was planned because of a recurrent stress urinary incontinence diagnosis, but a urethrovaginal fistula was found immediately before the procedure. After removing woven polyester, the previous sling material, simple fistula closure was carried out but failed. Usage of a vaginal speculum and powerful medical lamps during a stress test revealed leakage from both the urethrovaginal fistula and the external urethral meatus. She underwent another fistula closure using a Martius flap. Subsequently, a 1‐h pad test improved from 195 to 5.1 g/h. The remaining mild stress urinary incontinence did not necessitate further treatment.

**Conclusion:**

Anti‐incontinence procedures using synthetic materials can cause urethrovaginal fistula. Attention must be paid to the possibility of urethrovaginal fistula when patients complain of worsened incontinence postoperatively.

**Abbreviations & Acronyms**
MUS: mid‐urethral sling
SUI: stress urinary incontinence
UVF: urethrovaginal fistula

**Keynote message**

UVF is a rare complication which can be caused by anti‐incontinence procedures using synthetic materials. Medical practitioners must pay attention to the possibility of UVF when patients suffer from worsened incontinence following SUI surgery.

## Introduction

Bone anchor sling had been carried out as a less invasive option to treat SUI until its kits (ProteGen^®^, precut woven polyester sling; Microvasive/Boston Scientific Corp., Natick, MA, USA) were recalled from the market in 1999 due to complications such as vaginal exposure and pubic osteomyelitis.^1^, ^2^ As ProteGen^®^ was not approved by the Japanese Government, Japanese surgeons cut Hemashield^®^ (flat sheets of woven polyester; Microvasive/Boston Scientific Corp.) into similar shapes as ProteGen^®^ and used them in bone anchor sling in the late 1990s.^3^, ^4^ We experienced UVF with a delayed diagnosis after undergoing a bone anchor sling.

## Case presentation

A 69‐year‐old woman was referred to us due to a diagnosis of recurrent SUI while coughing, sneezing, and walking. Her incontinence required her to change 200cc urinary pads several times per day. Eleven years earlier, she underwent a bone anchor sling to treat SUI at another hospital. After the surgery, urinary incontinence was relieved completely, but it recurred 2 years prior to the referral.

A stress test in the lithotomy position confirmed leakage when coughing and straining. Although the leakage appeared to issue from the vagina at first, the second round of coughing showed leakage from the external urethral meatus. Retrospectively, coexistent UVF was overlooked as the lighting was poor and a vaginal speculum was not used. A 1‐h pad test resulted in 166 g of leakage. Uroflowmetry was normal (maximum flow rate 34 mL/s, voided volume 331 mL, and residual volume 0 mL). Videourodynamic study verified leakage and the bladder neck opening while coughing and straining without detrusor overactivity.

**Fig. 1:**
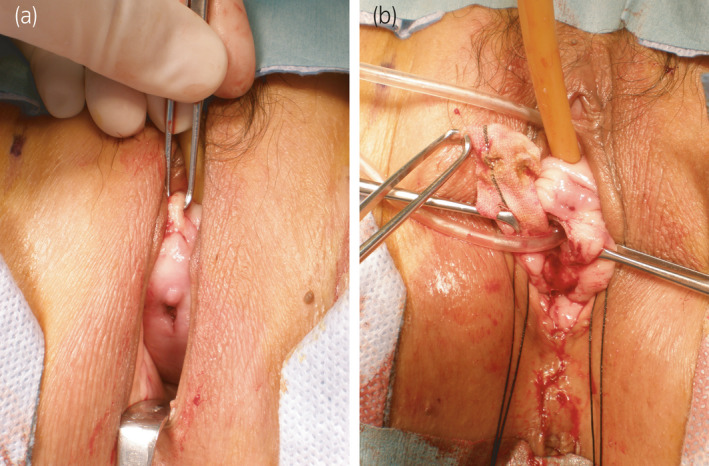
(a) A UVF, 5 mm in diameter, was discovered 2 cm proximal to the external urethral meatus in the anterior vaginal wall. A vaginal speculum and an Allis forceps were used allowing a clear view of the UVF. (b) Hemashield^®^, the previous sling material, was found in a folded condition under the fistula and removed entirely. A Nelaton catheter was inserted into the UVF and external urethral meatus.

An MUS was planned, but immediately before the procedure, a UVF was found 2 cm proximal to the external urethral meatus (Fig. 1). After Hemashield^®^, the previous sling material was removed entirely, the fistula was closed with absorbable sutures.

**Fig. 2:**
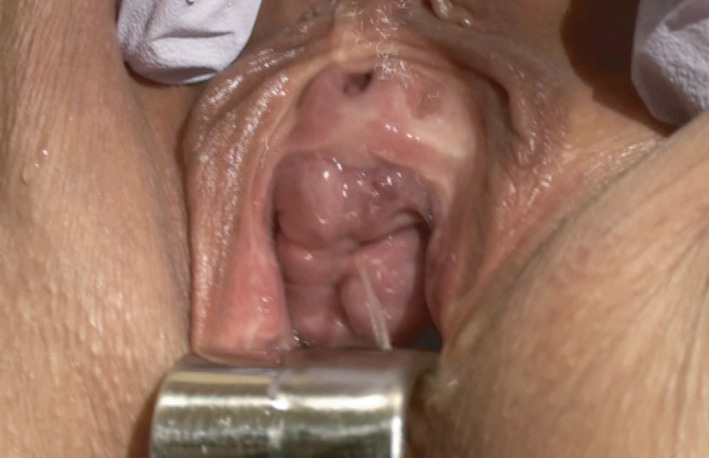
Usage of a vaginal speculum and powerful medical lamps during a stress test made it obvious that more leakage occurred from the recurred UVF (the same location, 2 mm in diameter) than from the external urethral meatus.

Urinary incontinence improved temporarily after the fistula closure but recurred rapidly. Three months later, a 1‐h pad test resulted in 195 g of leakage. Usage of a vaginal speculum and powerful medical lamps during a stress test made it obvious that more leakage occurred from the UVF than from the external urethral meatus (Fig. 2).

**Fig. 3:**
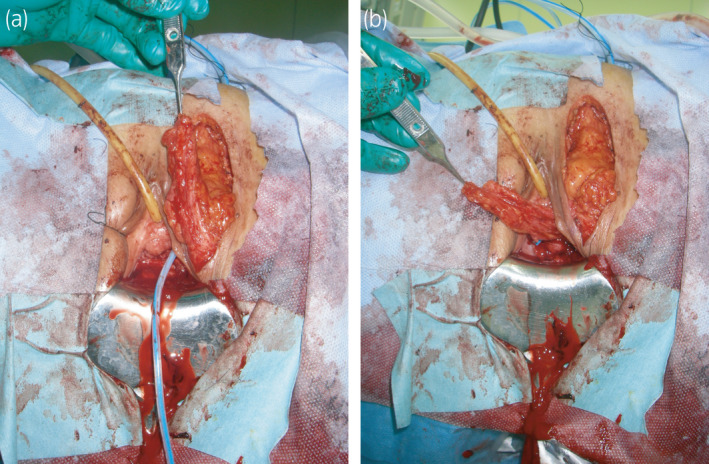
Second UVF closure with a Martius flap. (a) A skin incision was made in the left labium. The Martius flap was made while maintaining an adequate blood supply (monitored with Doppler ultrasound). (b) A tunnel was created between the left labium and the vaginal incision. The Martius flap was inserted into the tunnel and interposed between the urethra and the vaginal wall.

Subsequently, 6 months after the first closure, the patient underwent another fistula closure using a Martius flap (labial bulbocavernosus muscle/fat flap) (Fig. 3). Urinary incontinence markedly improved, a 6‐month postoperative stress test was negative, and a 1‐h pad test resulted in 5.1 g of leakage. She did not want further treatment for the remaining mild SUI.

## Discussion

While most UVFs occur following obstructed labor in developing countries, they are usually iatrogenic in developed countries.^5^ Common causes include surgeries for urethral diverticula and gender reassignment, radiotherapy for urogenital tumors, usage of pessaries for pelvic organ prolapse, and usage of indwelling urethral catheters (especially in patients with neurologic impairment).^5^, ^6^ Anti‐incontinence procedures using synthetic materials have also been reported to cause UVF. Bone anchor sling had been carried out in the 1990s until its kits (ProteGen^®^, woven polyester sling) were recalled due to vaginal exposure and osteomyelitis.^1^, ^2^ Kobashi *et al*. reported a case series of 34 patients who required the removal of woven polyester slings.^1^ Of those patients, 17 (50%) had vaginal exposure only, 7 (20%) had urethral exposure only, and 6 (17%) had UVF. In Japan, Kato *et al*. reported that 4/19 (21%) patients who underwent bone anchor sling procedures necessitated the removal of woven polyester slings due to vaginal exposure.^4^ In this report, we describe a case of UVF due to a woven polyester sling. This case had an extremely late onset of 11 years. Diagnosis of UVF was delayed due to co‐occurring recurrent SUI.

Symptoms of UVF largely depend on the size and location of the fistula.^5^ Although a large UVF can cause continuous leakage, a small proximal UVF may result in leakage with abdominal pressure. A distal UVF beyond the sphincteric mechanism may be asymptomatic or associated with postmicturition dribble due to urine accumulation in the vagina during voiding (vaginal reflux). In our case of proximal UVF, leakage occurred while coughing, sneezing, and walking, and thus it was difficult to differentiate UVF symptoms from recurrent SUI.

UVF can often be diagnosed with vaginal examination. In our case, we initially overlooked the coexisting UVF. During a stress test after a failed closure, usage of a vaginal speculum and powerful medical lamps enabled us to discover leakage from both the UVF and the external urethral meatus. Additionally, voiding cystourethrography, flexible cystoscopes, and 3D magnetic resonance imaging/computed tomography are reported to be useful when diagnosing UVF.^5^

Regarding UVF repair, a vaginal approach is the preferred option. Goodwin *et al*. reported that a vaginal approach achieved a better success rate (70% on the first attempt, 92% on the second attempt) than an abdominal approach (58%).^7^ Surgical principles are the same as those of vesicovaginal fistula repair: identifying the fistula, creation of a dissection plane between vaginal wall and urethra, watertight closure of urethral wall, interposition of tissue if needed, and closure of vaginal wall. The Martius flap has been reported to be an important adjunctive measure in the treatment of genitourinary fistulae.^8^, ^9^ After the failure of our first simple closure, we utilized the Martius flap successfully in the second closure.

Whether SUI surgery should be carried out concomitantly with fistula repair is a controversial topic. Although some authors argue that SUI and UVF should be repaired simultaneously,^10^ others argue fistula repair should be carried out first and then that the necessity of SUI surgery is evaluated.^11^ In our case, the patient was satisfied with the improvement of urinary incontinence after fistula repair and did not want further SUI surgery.

Despite being an obsolete procedure, bone anchor sling using woven polyester can cause vaginal/urethral exposure and UVF long after the procedure. Even the present gold‐standard MUS using polypropylene mesh can cause similar complications.^9^, ^12^ Therefore, we should be cautious about UVF when patients complain of worsened incontinence after SUI surgery.

## Author contributions

Kumiko Kato, Akitaka Suzuki: conception and design, acquisition of data, analysis and interpretation of data, drafting of the manuscript. Yuji Hayashi, Aika Matsuyama, Hiroki Sai, Akinobu Ishiyama, Takashi Kato, Satoshi Inoue: acquisition of data. Hiroki Hirabayashi, Shoji Suzuki: analysis and interpretation of data, supervision.

## Conflict of interest

The authors declare no conflict of interest.

## Approval of the research protocol by an Institutional Reviewer Board

Not applicable.

## Informed consent

Written informed consent was obtained from the patient.

## Registry and the Registration No. of the study/trial

Not applicable.
